# Beyond ordinal scales: making animal welfare count in policy analysis

**DOI:** 10.3389/fvets.2025.1556475

**Published:** 2025-03-06

**Authors:** Bob Fischer

**Affiliations:** Department of Philosophy, Texas State University, San Marcos, TX, United States

**Keywords:** animal welfare, benefit cost analysis (BCA), policy analysis, economics, ratio scale

## Abstract

Animal welfare is typically assessed using ordinal scales. That is, standard welfare assessment tools rank conditions relative to one another without claiming that one condition is worse than another by some specific magnitude. However, there are some practical purposes for which ordinal scales are insufficient, such as accounting for animal welfare in policy analysis. Here, I argue that insofar as we want standard policy analysis tools to capture impacts on animal welfare in a way that is scope sensitive—that is, in a way that properly recognizes differences in the number of animals affected—we need ways of representing animal welfare on ratio scales, not merely ordinal ones. Then, I briefly explain how some economists, who play important roles in policy analysis, are beginning to do this without the assistance of animal welfare scientists, veterinarians, and others. So, this perspective article serves as a call to those stakeholders, inviting them to collaborate with economists and policy analysts to improve existing methods or develop better alternatives that meet current needs.

## 1 Introduction

Animal welfare is typically assessed using ordinal scales. That is, standard welfare assessment tools rank conditions relative to one another without claiming that one condition is worse than another by some specific magnitude. In Mellor (1), for instance, which explains how to apply the Five Domains Model of animal welfare, “animal welfare challenges” are given grades from A (“none”) to E (“very severe”). But there is no quantitative answers to the question: How much worse is it to have an E-grade challenge compared to a D-grade challenge? Likewise, the framework takes no stance on whether the welfare benefit associated with moving an animal from an E-grade challenge to a D-grade challenge is the same as the one associated with moving an animal from a C-grade challenge to a B-grade challenge.

Granted, there are frameworks that appear to use ratio scales, such as the Welfare Quality animal welfare assessment system, which assigns scores from 0 to 100 to animal welfare across four domains (2). However, when it comes to producing overall scores, Blokhuis et al. do not simply average these numbers (indeed, they explicitly reject that possibility; Welfare Quality 2009). Instead, they take a weighted sum of the scores to prevent high scores in some domains from offsetting low scores in others. Then, they introduce four categories—“excellent,” “enhanced,” “acceptable,” and “not classified” (unacceptable)—and map the aggregate scores to those categories. Again, the quantitative relationships between these four categories are (intentionally) left unspecified.

This is appropriate for many practical purposes. For instance, if the question is whether some intervention is required, then a threshold on an ordinal scale can provide an answer. That is, a farm might have a policy that says: “Monitor B-grade animal welfare challenges and intervene to mitigate C- and higher-grade challenges.”

However, there are some practical purposes for which ordinal scales are insufficient, at least given certain plausible assumptions. Here, I consider one such purpose: namely, accounting for animal welfare in policy analysis. Policy analysis tools are methods for assessing the merits of competing policy proposals. Some standard policy analysis tools require that relevant considerations are represented in monetary terms (3); however, animal welfare typically is not represented that way; so, animal welfare is often either omitted or merely mentioned without being integrated into the analysis (4).

Here, I argue that insofar as we want standard policy analysis tools to capture impacts on animal welfare in a way that is sensitive to the number of animals affected, we need ways of representing animal welfare on ratio scales, not merely ordinal ones. Then, I briefly explain how some economists—who play important roles in policy analysis—are beginning to do this without the assistance of animal welfare scientists, veterinarians, and others. So, this perspective article serves as a call to those stakeholders, inviting them to collaborate with economists and policy analysts to improve existing methods or develop better alternatives that meet institutional needs.

## 2 Benefit–cost analysis

The aim of this section is to introduce benefit–cost analysis (BCA), which is a policy analysis tool on which many jurisdictions rely. While the central point of this perspective article could be made using other tools, such as cost-effectiveness analysis, BCA is sufficiently common and influential to be worth our attention.

In the US, BCA has been required for major federal regulations since the Reagan administration (Executive Order 12291 in 1981, later modified by Clinton’s Executive Order 12866). The UK has also been using BCA extensively since the 1980’s, the influence of which expanded under the Thatcher government. Finally, the EU has required impact assessments for major policy initiatives since 2002—assessments that are broader than BCA but often still include it.

The key feature of BCA is that, insofar as possible, all benefits and costs are expressed in monetary terms (i.e., “monetized”), allowing them to be compared on a common scale. This is the central appeal of BCA. By contrast, at the conclusion of a multi-criteria decision analysis—i.e., one that does *not* compare all benefits and costs on a common scale—we are left with difficult questions about how to balance the various pros and cons of a policy. In a BCA, it is clear whether the benefits outweigh the costs or vice versa, at least insofar as we are confident that all benefits and costs have been captured, and their values accurately represented, in the process.

This last point is the main concern with BCA: we may *not* be confident that all benefits and costs have been captured, and their values accurately represented, in the process. One prominent reason for this is that some benefits and costs are naturally expressed in monetary terms; others aren’t. This problem is not specific to animal welfare. For instance, when considering a piece of environmental regulation, the costs of compliance and enforcement are readily estimated; the benefits of cleaner air and the preservation of old-growth forest, by contrast, are not.

Economists appreciate this concern and, in response, have developed methods for monetizing “non-market goods” (i.e., goods that aren’t bought and sold on a market, as material goods and labor are, and so have no market price). These methods apply to the good of interest here—namely, animal welfare—and involve estimating how much stakeholders are willing to pay for the good in question. By using estimates of willingness to pay for non-market goods as estimates of their value, these goods can be compared directly with traditional economic considerations.

## 3 The problem of scope insensitivity and a solution

In general, we should expect larger benefits to be preferable to smaller ones, and thus more valuable. However, studies of stakeholder willingness to pay reveal that many stakeholders are scope insensitive: that is, their willingness to pay does not scale with the number of individuals affected. Desvousges et al. (5) provides the classic example of this phenomenon, where they found that consumers were willing to pay very similar amounts to prevent the deaths of 2,000, 20,000, and 200,000 migratory waterfowl. As a result, if consumer willingness to pay were used as the metric of the value of preventing these deaths, a standard method for ranking policies would be insensitive to differences in the number of animals affected by different options. For this reason, while the degree to which people are scope insensitive is a matter of some debate [see, e.g., (6, 7)], the phenomenon itself is well-attested and, when present, is generally viewed as a limitation of willingness to pay studies in policy analysis (8).

This is particularly concerning when it comes to valuing farmed animal welfare, as farmed animal populations are very large. A policy change affecting broiler production, for example, might affect tens of millions of animals. So, if stakeholders are scope insensitive, then their valuations are unlikely to reflect the significance of improving the welfare of so many individual animals.

There is a standard solution to this problem from health economics. In health economics, valuations of *human* welfare are not incorporated into BCA by asking stakeholders how much they are willing to pay to avert harms to some population by implementing some policy (e.g., averting deaths from a pandemic by implementing distancing requirements). Instead, health economists have methods for assigning a value to a single unit of human welfare, such as a “disability-adjusted life year” (DALY) (9). The DALY is a way of representing negative impacts on both the quantity and quality of life on a single scale, making it possible to compare health conditions that affect people differently: some significantly shorten life; others have smaller impacts on lifespan but major impacts on the quality of life. Given this unit, health economists can estimate the number of DALYs that would be averted by a policy change, multiply that number by the value of averting a DALY (estimated via surveys and observations of human choices), and produce a figure that represents the benefit of the relevant policy. So, if we suppose that, for the purposes of BCA, we should assume that the value of averting a DALY is $100,000, then a policy that averts 1,000 DALYs (e.g., by preventing life-shortening and painful respiratory conditions) has a value of $100,000,000. This method ensures that the overall value assigned to helping people is sensitive to the number of people who would be benefitted and the amount that they would be benefitted.

Similar methods are emerging to address this problem in the valuation of animal welfare. These methods allow economists to extrapolate the value of improving animal welfare in a way that is strictly scope-sensitive, where there is a one-to-one relationship between the number of animals affected and the overall valuation (10). As in the human case, these methods involve setting a baseline monetary value for improving the welfare of a single animal by a specific amount (again, estimated via surveys and observations of human choices); then, they produce a total valuation by multiplying by the number of animals affected [e.g., (11, 12)].

Crucially, though, these methods depend on being able to treat animal welfare the way we treat human well-being: namely, like a fungible quantity that we can aggregate across individuals. That is, these methods depend on being able to say that a given welfare improvement for one animal is equivalent to some magnitude change in welfare, which can be treated as equivalent to any other change of the same magnitude. Then, that magnitude can be multiplied by the number of individuals to estimate the impact of a particular policy and compare it to other policy options. Performing these kinds of calculations with welfare makes sense if we can measure welfare on a ratio scale. If, for instance, welfare is assessed on a 0 to 100 scale, then the difference between improving welfare 10 units from any point in the scale is directly comparable to any other such 10-unit improvement. Accordingly, let us suppose, just for illustrative purposes, that standard methods for estimating stakeholder willingness to pay support treating the value of improving one chicken’s welfare for a year by 10 units as being worth $1. Then, these new methods imply that, for the purpose of constructing a benefit–cost analysis, we should treat the value of improving 1,000 chickens’ welfare by the same amount—regardless of the specific welfare improvement—as being worth $1,000.

## 4 Economists’ adaptations of standard welfare assessment tools

Again, though, standard welfare assessment tools do not treat welfare like a quantity that we can aggregate across individuals. That is, these methods do not treat a given welfare improvement for one animal as equivalent to a fungible “amount” of welfare [there are exceptions—e.g., (13–15)—but these methods are far less influential].

As a result, economists are trying to adapt standard welfare assessment tools to suit the needs of BCA. For instance, Bennett et al. (16) tries to develop cardinal scores using the Welfare Quality framework, Espinosa (12) does the same thing for welfare conditions using the Five Freedoms, and Budolfson et al. (11) attempt this using the Five Domains. While the differences are important, we can use Espinosa’s (12) Five-Freedom Fulfillment Index (5FFI) as a representative example.

The 5FFI begins with the Five Freedoms and then defines five violation levels, ranging from none (0 points) to very severe (4 points). Then, it defines a method for producing an overall welfare score on a -⅓–1 scale. According to the 5FFI, an overall welfare score of 1 is perfect welfare (in the sense that there aren’t any violations of the Five Freedoms) while an overall welfare score of 0 is equivalent to severe violations (3 points) across all Five Freedoms (“33,333”). (By not making 0 equivalent to *very* severe violations across all Five Freedoms—“44,444”—the 5FFI can capture welfare states that are negative overall. These are states worse than death where animals ought to be euthanized.) Then, the overall welfare score for an animal is 1 − (the sum of all violation points)/(15). For the purposes of BCA, the 5FFI treats this -⅓–1 scale as a ratio scale. The difference between 0.1 and 0.2 is the same as the difference between 0.9 and 1. So, the 5FFI allows statements like, “An overall welfare score of 0.8 is 4x better than an overall welfare score of 0.2.” Moreover, it allows all welfare impacts to be aggregated to estimate the total welfare impact of a policy on a population of animals.

As should be obvious, there is nothing particularly complex about the 5FFI. It would be easy to define any number of similar frameworks using other standard welfare assessment tools. Moreover, the researchers behind standard welfare assessment tools are aware of this possibility. Indeed, many of them have intentionally chosen *not* to develop frameworks of this kind, as such simple approaches make a host of contentious methodological and ethical assumptions. For instance, while it is not obvious how to trade off the different Freedoms, the 5FFI simply treats them symmetrically. In addition, we might be uncertain about whether moving animals from “moderate” violations (2 points) to “mild” violations (1 point) makes the same difference to their welfare as moving them from “very severe” violations (4 points) to “severe” violation (3 points). Nevertheless, the 5FFI treats these changes as equivalent. So, since standard welfare assessment tools can serve many important purposes outside policy analysis without making such assumptions, there are good reasons to use them in those contexts.

As we have seen, however, standard welfare assessment tools are not well-suited to all important purposes. In particular, they are not appropriate for incorporating animal welfare into BCA in a way that is sensitive to the number of animals affected by policies. Hence, economists will continue to develop ways to represent welfare improvements on a ratio scale to address this issue.

## 5 The opportunity to improve economists’ adaptations

Animal welfare scientists and veterinarians have valuable knowledge about the relative severity of conditions that affect animals. Because they have deep knowledge of their species and have carefully observed these animals in a range of husbandry contexts, these individuals are well-positioned to judge how well off animals are, how much better off they can be, and which factors would make the largest difference to their welfare. As a result, animal welfare scientists and veterinarians have already provided many important insights into the measurement of animal welfare, developing frameworks that have been essential to understanding how best to improve the conditions of companion, farmed, research, and wild animals.

However, there remain purposes for which standard welfare assessment tools are inadequate. To integrate animal welfare into BCA in a way that properly reflects the number of animals affected by policies, we need the emerging methods that economists are developing. These methods involve setting a baseline valuation for improving the welfare of the average animal in a population by a specific amount; then, they calculate a total valuation by multiplying the baseline by the number of animals affected. This requires treating welfare as though it can be measured using standardized units that we can aggregate.

It is reasonable to object to decision-making tools that require treating welfare this way. For instance, someone might argue that the welfare impacts of minor and extreme suffering (e.g., the pain due to a small laceration vs. the pain due to botched slaughter) cannot be reduced to two numbers, however far apart they might be. Relatedly, someone might contend that such a quantitative framework obscures important differences between welfare impacts, such as acute but brief suffering and low-level but long-lasting stress. In the framework required for BCA, there is some amount of low-level but long-lasting stress that will be represented by the same number as acute but brief suffering, even though these are clearly very different sorts of experiences. With these sorts of objections in mind, animal welfare scientists might see themselves as contributing to a false analytical paradigm were they to help economists develop a framework that is suited to the constraints of BCA. From this perspective, the framework would present difficult, messy decisions as though they were simple empirical decisions that can be made by summing scores.

There are two ways to respond to such concerns. The first is to flag that welfare assessment frameworks have different purposes. One of them is to characterize a welfare state as accurately as possible. Another is to facilitate decision-making. And quite often, these purposes require developing entirely different frameworks. Consider, for instance, the World Health Organization’s Interagency Integrated Triage Tool (IITT), which categorizes (human) patients as red (high acuity; need to be seen immediately), yellow (moderate acuity; need to be seen soon), or green (low acuity; can wait) (17). This tool certainly obscures important differences between welfare states. However, that is no objection to the IITT: its purpose is to capture critical information about human welfare in a way that lends itself to the needs of particular decision-makers; relative to that purpose, its shortcomings are virtues, as all the information it obscures would be distracting in the relevant decision context. Likewise, for the kinds of macro-level decisions that economists are trying to inform, the relevant information is very coarse-grained.

Second, whatever the merits of any objections to a particular decision-making tool, it is essential to consider whether its use is an open question. If there are alternatives, then it can make sense to push for them. However, BCA is deeply entrenched in existing regulatory structures and the larger tradition of policy analysis. So, the choice for animal welfare scientists and veterinarians is not whether policy analysts employ BCA, but rather whether to facilitate the inclusion of animal welfare into an established policy analysis methodology. And while this process must attend to the constraints that economists face, it will be better insofar as it involves people with the expertise required to make these decision-making tools as accurate and impactful as possible.

## 6 Conclusion

Animal welfare scientists and veterinarians can be invaluable contributors to the task of representing animal welfare in policy analysis. Because the community has not prioritized this project, little effort has been devoted to identifying the various possible methods, confirming their suitability for policy analysis, and weighing their relative advantages and disadvantages. So, there is ample room for animal welfare scientists and veterinarians to collaborate with economists, policy analysts, philosophers, and others in this transdisciplinary project. By working together, it should be possible to move toward consensus-generating proposals about how best to represent animal welfare on a ratio scale.

## Data Availability

The original contributions presented in the study are included in the article/supplementary material, further inquiries can be directed to the corresponding author.
